# Assessing the Theoretical Efficacy of Combination Therapy Against Gram-Negative Infections in Neutropenic Pediatric Cancer Patients: Insights from the Statistical Analysis of Survey Data

**DOI:** 10.3390/antibiotics13121160

**Published:** 2024-12-02

**Authors:** Elio Castagnola, Francesca Bagnasco, Alessio Mesini, Philipp K. A. Agyeman, Roland A. Ammann, Marta Arrabito, Fabianne Carlesse, Maria Rosaria D’Amico, Giovanna Giagnuolo, Gabrielle M. Haeusler, Evgeny A. Idelevich, Christa Koenig, Thomas Lehrnbecher, Marie Luckowitsch, Mariaclaudia Meli, Giuseppe Menna, Giovanna Russo, Maria Elena Santolaya de Pablo, Arne Simon, Galina Solopova, Lillian Sung, Annalisa Tondo, Andreas H. Groll

**Affiliations:** 1 Infectious Diseases Unit, IRCCS Istituto Giannina Gaslini, 16147 Genoa, Italy; eliocastagnola@gaslini.org; 2 Epidemiology and Biostatistics Unit, Scientific Directorate, IRCCS Istituto Giannina Gaslini, 16147 Genoa, Italy; francescabagnasco@gaslini.org; 3 Division of Pediatric Infectious Disease, Department of Pediatrics, Inselspital, Bern University Hospital, University of Bern, 3010 Bern, Switzerland; philipp.agyeman@insel.ch; 4 StatConsult Ammann, 3400 Burgdorf, Switzerland; info@statconsultammann.ch; 5 Hematology-Oncology Unit, AOU Policlinico “G. Rodolico-San Marco”, Department of Clinical and Experimental Medicine, University of Catania, 95123 Catania, Italy; marta-arrabito@hotmail.it (M.A.); mclaudiameli@gmail.com (M.M.); diberuss@unict.it (G.R.); 6 Pediatric Department, Federal University of Sao Paulo, Sao Paulo 04023-062, Brazil; fabiannecarlesse@gmail.com; 7 Oncology Pediatric Institute-IOP-GRAACC, Federal University of Sao Paulo, Sao Paulo 04023-062, Brazil; 8 Unità Operativa di Trapianto di Cellule Staminali Emopoietiche e Terapie Cellulari, Azienda Ospedaliera di Rilievo Nazionale, Santobono-Pausilipon, 80123 Naples, Italy; mrdamico@tiscali.it; 9 Unità Operativa di Ematologia, Azienda Ospedaliera di Rilievo Nazionale, Santobono-Pausilipon, 80123 Naples, Italy; g.giagnuolo@santobonopausilipon.it (G.G.); g.menna@santobonopausilipon.it (G.M.); 10 National Center for Infections in Cancer, Peter MacCallum Cancer Centre, Melbourne 3000, Australia; gabrielle.haeusler@rch.org.au; 11 Department of Infectious Diseases, Royal Children’s Hospital, Melbourne 3000, Australia; 12 Friedrich Loeffler Institute of Medical Microbiology, University Medicine Greifswald, 17475 Greifswald, Germany; evgeny.idelevich@med.uni-greifswald.de; 13 Institute of Medical Microbiology, University Hospital Muenster, 48149 Münster, Germany; 14 Division of Pediatric Hematology/Oncology, Department of Pediatrics, Inselspital, Bern University Hospital, University of Bern, 3010 Bern, Switzerland; christa.koenig@insel.ch; 15 Department of Pediatrics, Division of Hematology, Oncology and Hemostaseology, Goethe University Frankfurt, 60590 Frankfurt am Main, Germany; thomas_lehrnbecher@yahoo.com (T.L.); marie.luckowitsch@gmail.com (M.L.); 16 Department of Infectious Diseases Hospital Dt. Luis Calvo Mackenna, Faculty of Medicine, Universitad de Chile, Santiago 7500539, Chile; msantola@med.uchile.cl; 17 Klinik fur Paediatrische Onkologie und Hematologie, Universitaetklinikum des Saarlandes Homburg, 6642 Homburg, Germany; arne.simon@uks.eu; 18 Department of Infection Control, Dmitry Rogachev National Medical Research Center of Pediatric Hematology, Oncology and Immunology, Moscow 117997, Russia; 19 Pediatric Oncology Supportive Care, Division Hematology/Oncology, The Hospital for Sick Children, Toronto, ON M5G 0A4, Canada; lillian.sung@sickkids.ca; 20 Pediatric Hematology Oncology, Meyer Children’s Hospital IRCCS Florence, 50134 Florence, Italy; annalisa.tondo@meyer.it; 21 Infectious Diseases Research Program, Center for Bone Marrow Transplantation and Department of Pediatric Hematology/Oncology, University Children’s Hospital, 48149 Muenster, Germany; andreas.groll@ukmuenster.de

**Keywords:** febrile neutropenia, empiric antibiotic therapy, combination therapy, Gram-negative bacteriamia

## Abstract

**Background**: Empirical antibacterial therapy for febrile neutropenia reduces mortality due to Gram-negative blood stream infections (BSIs). Pediatric guidelines recommend monotherapy with an antipseudomonal beta-lactam or a carbapenem and to add a second anti-Gram-negative agent in selected situations. We evaluated the changes in the proportions of resistance of beta-lactam monotherapies vs. their combination with amikacin, and the possible impact on ICU admission or death. **Results**: 797 BSIs due to Gram-negative bacteria in 685 patients were included. Combination therapies with amikacin had a lower percentage of isolates resistant to one or to both drugs compared with the respective monotherapy. The highest OR for ICU admission was observed when both drugs of the combination of meropenem–amikacin were resistant. Mortality was significantly associated with relapse or the progression of the underlying malignancy, and resistance to both drugs of the combinations of cefepime–amikacin or meropenem–amikacin. **Methods**: This study was based on data collected for a large multinational study, in which the susceptibility of Gram-negative bloodstream isolates was categorized following either EUCAST or CLSI according to local laboratory standards. An escalation antibiogram was generated for each selected drug. For resistant bacteria, the conditional susceptibility probability on resistance was calculated. **Conclusions**: In pediatric cancer patients with Gram-negative BSIs, the proportion of the resistant organism correlates with ICU admission or death, which may be reduced by combination therapy. In patients with suspected or confirmed Gram-negative BSIs that are not-improving or deteriorating under monotherapy, escalation to meropenem may represent the best option. Amikacin should be preferred when combination therapy is considered with ciprofloxacin as an alternative in the case of impaired renal function.

## 1. Background

Febrile neutropenia is a medical emergency due to the high risk of life-threatening infections. The introduction of empirical antibacterial therapy in the latter half of the 20th century was a significant advancement, as studies showed that the prompt administration of broad-spectrum antibiotics in febrile neutropenic patients reduced the mortality associated with bloodstream infections (BSIs) caused by Gram-negative bacteria [1,2]. This treatment approach is now standard for both children and adults presenting with fever or suspected infection related to chemotherapy or conditioning regimens for hematopoietic cell transplantation (HCT) [3,4].

In pediatric patients, the incidence of BSIs during febrile neutropenia varies, typically reported at less than 20%, depending on the underlying disease and treatment phase [5,6,7]. Notably, Gram-negative bacteria account for approximately 50% of these BSI episodes [8,9]. Current international pediatric guidelines recommend initiating therapy with a monotherapy regimen—either an antipseudomonal beta-lactam (such as piperacillin-tazobactam or cefepime) or a carbapenem (predominantly meropenem) [3,10]. According to these guidelines, the initial addition of a second anti-Gram-negative agent, such as an aminoglycoside, should be reserved for clinically unstable patients, those with suspected resistant infections, or in centers with high rates of resistant pathogens. In such cases, the choice of initial therapy should be informed by local antibiotic resistance patterns [3,10]. With this strategy, the overall mortality rate in febrile neutropenia episodes was below 2% [6,7,11]. However, mortality rates approached 10% in cases of Gram-negative BSI, particularly in patients infected with antibiotic-resistant strains, which also heightened the risk of intensive care unit (ICU) admission [12]. 

Considering the poor outcomes associated with resistant Gram-negative infections, and the increasing rates of patterns of co-resistance [13], a multicenter study in adults proposed the development of an “escalation antibiogram” based on local epidemiological data. This antibiogram would guide modifications to empirical treatment, allowing for the addition of a new drug or switching to another drug or combination for patients not responding to first-line therapy, tailored to local antimicrobial susceptibility [14]. Recently, a Bayesian model was developed in a local epidemiology setting demonstrating encouraging results on small groups of patients [15].

Building on these considerations and utilizing data from a large multinational pediatric study on antibiotic susceptibility in Gram-negative BSIs [12], we first assessed the proportion of resistant isolates to recommended monotherapy agents (piperacillin–tazobactam, cefepime, ceftazidime, and meropenem). We also investigated whether the addition of amikacin influenced resistance rates and impacted ICU admission and mortality rates. In the next phase, we evaluated the most effective modification strategies (i.e., the addition of a second drug or the substitution of the initial therapy) and the potential implications of this escalation antibiogram for patients who were not responding or deteriorating while awaiting antibiotic susceptibility test results.

The primary objective of this analysis was to conduct a comprehensive assessment of the impact that incorporating a second antibiotic into existing treatment regimens has on the percentage of bacterial strains exhibiting resistance. In this study, we specifically defined resistant strains as those bacterial populations that demonstrate resistance to both the initially prescribed antibiotic and the subsequently introduced antibiotic.

Our evaluation aimed to explore whether the strategic combination of these two antimicrobial agents could effectively reduce the prevalence of such resistant strains within the tested population. We hypothesized that the addition of a second antibiotic might not only target different mechanisms of action but also enhance the overall therapeutic efficacy against infections caused by resistant organisms. By systematically analyzing resistance patterns and treatment outcomes, we sought to provide evidence-based insights into how this dual therapy could influence clinical practices and improve patient management in cases of antibiotic resistance.

## 2. Results

An analysis was performed on a total of 797 BSIs caused by Gram-negative bacteria in 685 patients and followed in 13 centers. Supplementary Table S1 provides the detailed etiologies of the episodes included. Neutropenia at the onset of BSI was present in 585 (73.4%) of the episodes.

### 2.1. Estimated Effect of Combination Therapy

Data on antibiotic susceptibility were not uniformly available for all the drugs, and non-tested isolates ranged from 17.0% for meropenem to 32.3% for cefepime (Supplementary Table S2). 

As summarized in Table 1, the proportion of isolates resistant to any single drug was <10% for meropenem and amikacin, while it ranged from 22 to approximately 30% for the cephalosporins. Combination therapy had a lower percentage of isolates resistant to one or to both drugs compared to the proportions of resistance in monotherapy, both in absolute and relative terms. This observation was particularly evident for piperacillin–tazobactam, cefepime, and ceftazidime, but was also noted for meropenem, although to a lesser degree (Table 1). Subsequently, analyses were performed to evaluate the role of combination therapy on ICU admission and mortality. Overall, ICU admission was needed in 15.1% (120/797) of the cases and death occurred in 8.1% (65/797). Table 2 reports the results of the multilevel mixed effects logistic regression model for ICU admission. The variables significantly associated with this outcome included the diagnosis of hematologic malignancy and the presence of neutropenia, and the highest OR was observed in the presence of resistance to both the drugs of the combination meropenem–amikacin. Noteworthily, lower but still significant risk was still present in the case of resistance to only one of these two drugs. The presence of a center-level variation was suggested both by the estimation of the random effect and the statistically significant *p*-value of the LR test comparing a multilevel mixed effects logistic model versus standard logistic regression (Table 2).

Table 3 reports the results of the multilevel mixed effects logistic regression model for mortality. Here, variables significantly associated with this outcome included the relapse/progression of the underlying disease, and resistance to both drugs of the combinations cefepime–amikacin or meropenem–amikacin, but not to any single drug of the combinations. Also, in this case, the center-level variance effect was not negligible (Table 3).

### 2.2. Escalation Antibiogram

Included in this analysis were only the 382/797 (47.9%) Gram-negative isolates (detailed in Supplementary Table S3) for which antimicrobial susceptibility was available for all the six antibiotics considered, and the results are summarized in Table 4. 

**Figure 1:**
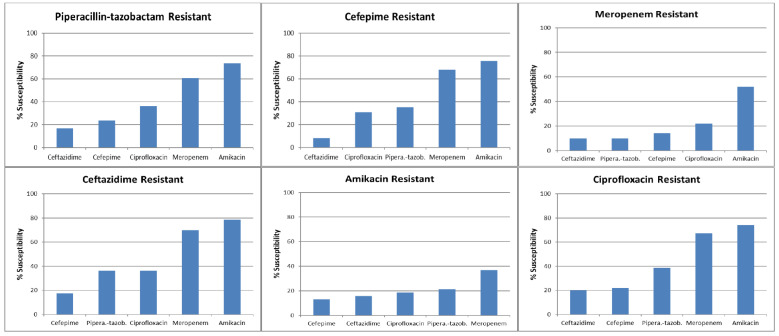
Escalation antibiogram-susceptibility proportions among the subset of Gram-negative isolates resistant to an antibiotic agent of interest; molecules are shown in ascending order according to susceptibility percentage.

Figure 1 depicts the proportion of isolates susceptible to the other antibiotics in the presence of resistance to a given drug, with detailed data provided in Table 4. Amikacin was confirmed as the most useful “companion” drug for all the beta-lactams, at least in the study settings, with a proportion of susceptible isolates that was always higher than that of meropenem. On the other hand, meropenem represented the antibiotic of choice for treatment shift when piperacillin–tazobactam, ceftazidime, or cefepime were resistant. Of note, the susceptibility of ciprofloxacin was <40% in all the conditions of resistance to other antibiotics, including amikacin.

## 3. Discussion

We conducted a comprehensive analysis examining the antimicrobial susceptibility profiles of 797 Gram-negative bacterial isolates from bloodstream infections (BSIs) in pediatric patients undergoing chemotherapy or hematopoietic cell transplantation (HCT) [12]. The goal was to assess the impact of moving from monotherapy to combination therapy—specifically, a beta-lactam combined with amikacin—on resistance levels and to evaluate the potential for reducing ICU admissions or mortality in neutropenic children. The findings underscore that combination therapy can effectively reduce the incidence of treatment failure linked to antibacterial resistance among Gram-negative isolates in BSI. Moreover, the study showed that resistant infections were associated with an elevated risk of ICU admission and mortality.

Empirical antibacterial therapy for febrile neutropenia has been adopted to provide early intervention against potential Gram-negative BSI in neutropenic patients, who are known to have a heightened risk of mortality [1,2]. Contemporary international pediatric guidelines continue to endorse this approach by recommending monotherapy with an antipseudomonal beta-lactam or carbapenem as the standard regimen [3,10]. Combination therapy, on the other hand, is typically reserved for patients exhibiting clinical instability, in cases where a resistant infection is suspected, or in healthcare settings where high rates of resistant pathogens are common [3,10]. Our study supports the recommendation to avoid meropenem as a first-line treatment; instead, it suggests reserving meropenem as a critical “rescue” therapy for cases of Gram-negative bacteremia that fail to respond to initial beta-lactam monotherapy. Additionally, our findings reinforce the importance of tailoring empirical therapy for febrile neutropenia based on local epidemiology, which is likely the most crucial recommendation today.

One relevant meta-analysis on the effectiveness of empirical febrile neutropenia therapy in pediatric oncology patients found no additional benefit in adding amikacin to beta-lactams to prevent treatment failures compared to monotherapy [16]. Most febrile neutropenia episodes in pediatric patients are classified as fever of unknown origin [5], and Gram-negative BSIs are confirmed in only a subset of these cases, with an overall low mortality rate [6,7,11]. Therefore, current recommendations are largely based on inferences drawn from febrile neutropenia trials rather than specific evidence targeting the treatment of Gram-negative BSI in neutropenic children [1,2]. Furthermore, randomized clinical trials investigating empirical therapy for febrile neutropenia have yet to be parameterized to assess the efficacy of treatments against BSI—particularly Gram-negative BSI—or to measure their impact on all-cause mortality. We believe that our findings provide valuable insights that should be considered in the ongoing development of treatment strategies and clinical guidelines for these complex infections.

Our study also offers actionable information for managing cases in which a pediatric patient with Gram-negative BSI receiving monotherapy fails to improve or shows signs of deterioration before susceptibility test results are available. A potential solution could involve the development of an “escalation antibiogram” [14], where transitioning to meropenem may offer the most effective shift in therapy. Adding amikacin as part of combination therapy could be considered; although neither meropenem nor amikacin achieved 100% efficacy, their combined use may enhance therapeutic outcomes. These results are consistent with findings from recent studies utilizing the “weighted-incidence syndromic combination antibiogram” approach for Gram-negative BSI in pediatric oncology populations [17]. Though tobramycin and tigecycline are also used in combination regimens for febrile neutropenia and in escalation antibiograms [14,18,19], limited susceptibility data for these agents prevented their inclusion in our analysis. Ciprofloxacin, despite offering a similar spectrum of activity to amikacin and being suitable for patients with renal impairment, exhibited high resistance rates in our analysis and may therefore not be suitable for empirical escalation therapy.

## 4. Patients and Methods

The analysis was performed on data collected between 2015 and 2017 during a large multinational study on BSI in pediatric patients receiving antineoplastic chemotherapy or conditioning regimens for HCT [12]. In this study, pathogens were categorized as susceptible or resistant to antibiotics according to the local microbiology laboratory classifications following the European Committee on Antimicrobial Susceptibility testing (EUCAST) [http://www.eucast.org, 28 June 2024] or the Clinical and Laboratory Standards Institute (CLSI, Berwyn, PA, USA) [https://clsi.org, 28 June 2024] definitions available at that time. This approach was chosen as the minimum inhibitory concentrations were not consistently available. In the cases of intermediate or dose-dependent susceptibility, the strain was recorded as susceptible, based on the assumption that maximal drug dosages were administered. Antibiotics for whom susceptibility was collected were chosen based on the following considerations:

- Piperacillin–tazobactam, ceftazidime, cefepime, and meropenem are recommended as monotherapy for initial empirical therapy of febrile neutropenia according to different guidelines [3,10,20,21]. In addition, ceftazidime resistance could represent a surrogate for the presence of extended spectrum or AmpC beta-lactamases [22];
- Amikacin is frequently included in combination therapy [20];
- Ciprofloxacin has a spectrum of anti-Gram-negative activity like that of amikacin and may represent a possible alternative to aminoglycosides, particularly in the presence of impaired renal function.

Data on the other drugs occasionally used in the trials of the empirical treatment of febrile neutropenia (e.g., doripenem, tobramycin, tigecycline) were not included, as they were not available or available only for a small number of cases, making an evaluation impossible for the purposes of the present study. No data were available on the susceptibility to more recently approved antibiotics that have extended activity against resistant Gram-negatives: ceftazidime–avibactam, ceftolozane–tazobactam, cefiderocol, meropenem–vaborbactam, imipenem–relebactam, intravenous fosfomicin, and aztreonam.

### Statistical Analysis

Resistance in Gram-negative bacteria was reported in terms of absolute and percentage values. The absolute reduction in proportions of resistant strains observed with combination therapy compared to monotherapy was calculated as the difference between the proportion of resistance in combination and the proportion of resistance in monotherapy (reference value). The percentage of relative reduction in proportions of resistant strains was calculated as the percentage of the ratio between the reduction in proportions of resistant strains and the reference value.

The association between binary outcome variables (ICU admission or death) and independent variables was assessed by multilevel (two levels) mixed effects logistic regressions [23], or by standard logistic regression and reported in terms of the odds ratio (OR) and 95% confidence interval (CI). The two-level model had one random-effect equation—a random intercept at the center level. The demographic, clinical characteristics of the patients at the time of the Gram-negatives BSI and the susceptibility of combination therapy with a beta-lactam and an amikacin were entered into the multivariable models. A likelihood ratio (LR) test was used to measure the effect of each predictor and to compare a multilevel mixed effects logistic model versus the standard logistic regression that was performed in case of a statistically insignificant LR test.

An escalation antibiogram was generated for each of the six selected drugs (meropenem, cefepime, piperacillin–tazobactam, amikacin, ceftazidime, and ciprofloxacin) considering Gram-negative isolates for which these antibiotics were simultaneously tested. Among Gram-negatives resistant to a given antibiotic, the conditional susceptibility probability on resistance, i.e., the likelihood of susceptibility to each of the other five agents, was calculated. The susceptibility percentages were shown among the subset of Gram-negative organisms resistant to an antibiotic agent of interest.

All the tests were two-tailed and a *p* value < 0.05 was considered statistically significant. All the analyses were performed using Stata (StataCorp. Stata Statistical Software, Release 16.0, College Station, TX, USA, StataCorporation, 2019).

## 5. Conclusions

The most important limitation of this study lies in its multicenter and retrospective nature, based on local epidemiology, although from several centers worldwide, with the potential for a lack of some information, like susceptibility to specific drugs (e.g., tobramycin), which is impossible to overcome. In particular, this study was conducted from 2015 to 2017 and at that time, amikacin served as the standard for treating Gram-negative BSI, including those due to *P. aeruginosa*. Most recent CLSI, but not EUCAST, (https://clsi.org/about/blog/ast-news-update-june-2023-new-clsi-m100-ed33-updated-aminoglycoside-breakpoints-for-enterobacterales-and-pseudomonas-aeruginosa/; https://www.eucast.org/fileadmin/src/media/PDFs/EUCAST_files/Breakpoint_tables/v_14.0_Breakpoint_Tables.pdf, checked on 21 February 2024) recommendations advise against the use of amikacin in systemic pseudomonal infections, introducing a shift in antibiotic practice compared to the routine in the period 2015–2017. Over the long period since the initial study, the epidemiology of antibiotic resistance has certainly evolved, with an increase in extended-spectrum beta-lactamase and carbapenemase-producing strains. As a result, the findings may not be directly applicable to today’s epidemiological landscape. This is certainly a weakness of this study. Nonetheless, our study could be considered as a “proof of concept”, suggesting that combining an aminoglycoside (that was amikacin in this study period and could be tobramycin today, at least for some centers) significantly enhances the spectrum of activity of empirical therapy of febrile neutropenia. In any case, the conclusions of this study remain valid, providing flexibility in aminoglycoside selection based on local epidemiological factors, despite changes in individual drug recommendations. Our results also indicate that the escalation antibiogram should be strictly based on local epidemiology, but, in general, aminoglycosides should be considered for combination, while, above all, our data provide further support for the recommendation to use meropenem very carefully (or not at all) as a standard first-line agent for empirical therapy, and to reserve it in the case escalation as needed. 

Data on the activity and efficacy of new antibiotics with enhanced activity against resistant Gram-negative organisms were not included in this study since these agents were not available at the time of data collection. However, information on these agents in the context of the empirical treatment of febrile neutropenia is still scant: ceftolozane–tazobactam was evaluated in a small randomized clinical trial (100 patients enrolled, 47 treated with the drug, 3 cases of Gram-negative bacteremia) with the reported clinical outcomes better than the standard of care therapy (cefepime, piperacillin–tazobactam, meropenem) [24], while ceftazidime–avibactam was used for febrile neutropenia in patients known to be infected/colonized with resistant isolates [25], but not in the “general population”. Finally, no data are available for clinical use in this indication for cefiderocol, meropenem–vaborbactam, imipenem–relebactam, and intravenous fosfomycin, and not all these agents are approved for use in children with a validated dosage recommendation.

The multilevel mixed effects logistic regressions showed an evident center-level variation on antibiotic resistance among Gram-negatives. This observation further stresses the necessity to design management strategies, not only based on static guideline recommendations, but to adapt them, as recommended, to the local epidemiology of the individual institution [3,10]. This should become the number one recommendation in future guidelines.

## Figures and Tables

**Table 1:** Changes in resistance proportions from monotherapy to combination of beta-lactam/carbapenem + amikacin among 797 Gram-negative isolates in 685 patients.

	Monotherapy		Beta-Lactam + Amikacin					
Drug	Susceptible #	Resistant #	Resistant to One Drug of the Combination §	Resistant to Both Drugs of the Combination §	Absolute Reduction in Percentages of Resistant Strains *		Relative Reduction in Percentages of Resistant Strains **	
					One drug	Both drugs	One drug	Both drugs
Piperacillin–tazobactam	50.9 (406)	21.8 (174)	20.3 (162)	4.5 (36)	−1.5	−17.3	−6.9	−79.4
Cefepime	41.9 (334)	25.8 (206)	21.3 (170)	6.0 (48)	−4.5	−19.8	−17.4	−76.7
Meropenem	74.0 (590)	9.0 (72)	7.5 (60)	4.5 (36)	−1.5	−4.5	−16.7	−50.0
Ceftazidime	46.0 (367)	29.5 (235)	25.0 (199)	6.0 (48)	−4.5	−23.5	−15.3	−79.7
Amikacin	61.9 (493)	7.5 (60)						

Data are reported as percentages (absolute numbers); #, the denominator of percentages is represented by the total of 797 Gram-negative isolates. Frequencies of non-tested isolates are reported in Supplementary Table S2; §, the denominator of percentages is represented by the total of 797 Gram-negative isolates. Frequencies of non-tested and of susceptible isolates are reported in Supplementary Table S2; *, absolute reduction = percentage of resistance in combination–percentage of resistance in monotherapy; **, relative reduction = (absolute reduction/percentage of resistance in monotherapy) × 100.

**Table 2:** Multilevel mixed effects logistic regression model for ICU admission in Gram-negative bloodstream infection.

Factors	OR 95%CI	*p*-Value
Gender, male vs. female	0.9 (0.5–1.4)	0.561
Age at bloodstream infections, years	1.0 (0.9–1.1)	0.421
Underlying disease		**0.025**
NMD vs. HM	0.7 (0.3–1.8)	
ST vs. HM	**0.4 (0.2–0.8)**	
Allogeneic stem cell transplant phase		0.322
Pre-engraftment vs. no allogenic-HSCT	1.1 (0.5–2.3)	
Acute GvHD vs. no allogenic-HSCT	2.8 (0.8–9.7)	
Chronic GvHD vs. no allogenic-HSCT Post-engraftment vs. no allogenic-HSCT	0.7 (0.1–3.6) 0.5 (0.1–1.7)	
Relapse/progression, yes vs. no	1.3 (0.7–2.3)	0.330
Neutropenia, yes vs. no	**2.0 (1.03–4.0)**	**0.034**
Previous antibacterial exposure (prophylaxis/therapy) ^1^		0.710
Fluoroquinolones vs. no one/β-lactams	0.3 (0.1–2.9)	
Standard regimen vs. no one/β-lactams	1.3 (0.7–2.3)	
Carbapenem vs. no one/β-lactams	1.4 (0.7–2.8)	
Combination ^2^ vs. no one/β-lactams Others vs. no one/β-lactams	1.3 (0.2–9.2) 1.4 (0.5–4.1)	
**Piperacillin–tazobactam + amikacin**		**0.503**
Resistant vs. susceptible	0.6 (0.1–3.0)	
Not tested/resistant to 1 antibiotic of the combination vs. susceptible	0.6 (0.3–1.4)	
**Cefepime + amikacin**		**0.464**
Resistant vs. susceptible	5.9 (0.2–145.1)	
Not tested/resistant to 1 antibiotic of the combination vs. susceptible	1.6 (0.6–4.6)	
**Meropenem + amikacin**		**0.003**
Resistant vs. susceptible	13.4 (2.6–68.2)	
Not tested/resistant to 1 antibiotic of the combination vs. susceptible	3.3 (1.3–8.3)	
**Ceftazidime + amikacin**		**0.058**
Resistant vs. susceptible	0.4 (0.1–10.6)	
Not tested/ resistant to 1 antibiotic of the combination vs. susceptible	0.3 (0.1–0.8)	
Random effect, estimated variance component at the center level	1.6 (0.5–5.1)	
LR test vs. logistic regression *, *p*-value	<0.001	

NMD = non-malignant disease receiving allogeneic stem cell transplant; ST = solid tumor; HM = hematologic malignancy; HSCT = hematopoietic stem cell transplantation; GvHD = graft vs. host disease; LR test = likelihood ratio test; boldface = statistically significant results; two-level mixed effects logistic regression with random effects for centers. * If *p*-value of LR test, comparing multilevel mixed effects logistic model versus standard logistic regression, was statistically significant, standard logistic regression was not performed. ^1^ β-lactams not active vs. *P. aeruginosa* was considered as reference group due to no observed events in this group.; ^2^ Combination of two or more of the following fluoroquinolone/β-lactams not active vs. *P. aeruginosa*/standard regimen active vs. *P. aeruginosa*/carbapenem.

**Table 3:** Multilevel mixed effects logistic regression model of Gram-negative bacteremia for mortality.

Factors	OR 95%CI	*p*-Value
Gender, male vs. female	0.6 (0.3–1.1)	0.124
Age at bloodstream infections, years	0.9 (0.8–1.0)	0.050
Underlying disease		0.081
NMD vs. HM	1.7 (0.7–4.7)	
ST vs. HM	0.4 (0.1–1.1)	
Allogeneic stem cell transplant phase		0.389
Pre-engraftment vs. no allogenic-HSCT	0.9 (0.4–2.5)	
Acute GvHD vs. no allogenic-HSCT	3.3 (0.7–16.2)	
Chronic GvHD vs. no allogenic-HSCT Post-engraftment vs. no allogenic-HSCT	2.6 (0.5–13.1) 2.3 (0.8–6.9)	
Relapse/progression, yes vs. no	3.7 (1.8–7.5)	<0.001
Neutropenia, yes vs. no	2.2 (0.9–5.4)	0.063
Previous antibacterial exposure (prophylaxis/therapy) ^1^		0.174
Fluoroquinolones vs. no one/β-lactams	0.6 (0.1–6.2)	
Standard regimen vs. no one/β-lactams	1.0 (0.4–2.4)	
Carbapenem vs. no one/β-lactams	2.2 (0.9–5.9)	
Combination ^2^ vs. no one/β-lactams Others vs. no one/β-lactams	4.5 (0.7–27.8) 2.8 (0.8–10.1)	
**Piperacillin–tazobactam + amikacin**		**0.470**
Resistant vs. susceptible	0.4 (0.1–2.4)	
Not tested/ resistant to 1 antibiotic of the combination vs. susceptible	0.5 (0.2–1.6)	
**Cefepime + amikacin**		**0.079**
Resistant vs. susceptible	30.2 (1.3–682.1)	
Not tested/ resistant to 1 antibiotic of the combination vs. susceptible	2.9 (0.8–10.2)	
**Meropenem + amikacin**		**<0.001**
Resistant vs. susceptible	27.2 (3.9–188.1)	
Not tested/resistant to 1 antibiotic of the combination vs. susceptible	1.6 (0.6–4.6)	
**Ceftazidime + amikacin**		**0.328**
Resistant vs. susceptible	0.1 (0.01–2.5)	
Not tested/resistant to 1 antibiotic of the combination vs. susceptible	1.0 (0.3–3.4)	
Random effect, estimated variance component at the center level	0.9 (0.2–4.5)	
LR test vs. logistic regression *, *p*-value	0.003	

NMD = non-malignant disease receiving allogeneic stem cell transplant; ST = solid tumor; HM = hematologic malignancy; HSCT = hematopoietic stem cell transplantation; GvHD = graft vs. host disease, LR test = likelihood ratio test; boldface = statistically significant results; two-level mixed effects logistic regression with random effects for centers. * If *p*-value of LR test, comparing multilevel mixed effects logistic model versus standard logistic regression, was statistically significant, standard logistic regression was not performed. ^1^ β-lactams not active vs. *P. aeruginosa* were considered as reference group due to no observed events in this group. ^2^ Combination of two or more of the following fluoroquinolone/β-lactams not active vs. *P. aeruginosa*/standard regimen active vs. *P. aeruginosa*/carbapenem.

**Table 4:** Antibiotic susceptibility for drugs evaluated for the possibility of the escalation antibiogram in 382 Gram-negative strains.

Antibiotic	Susceptible	Resistant
**Overall n = 382**		
Piperacillin–tazobactam	70.2 (268)	29.8 (114)
Cefepime	64.9 (248)	35.1 (134)
Meropenem	86.9 (332)	13.1 (50)
Ceftazidime	61.0 (233)	39.0 (149)
Amikacin	90.1 (344)	9.9 (38)
Ciprofloxacin	68.8 (263)	31.2 (119)
**Piperacillin–tazobactam resistant, n = 114**		
Cefepime	23.7 (27)	76.3 (87)
Meropenem	60.5 (69)	39.5 (45)
Ceftazidime	16.7 (19)	83.3 (95)
Amikacin	73.7 (84)	26.3 (30)
Ciprofloxacin	36.0 (41)	64.0 (73)
**Cefepime resistant, n = 134**		
Piperacillin–tazobactam	35.1 (47)	64.9 (87)
Meropenem	67.9 (91)	32.1 (43)
Ceftazidime	8.2 (11)	91.8 (123)
Amikacin	75.4 (101)	24.6 (33)
Ciprofloxacin	30.6 (41)	69.4 (93)
**Meropenem resistant, n = 50**		
Piperacillin–tazobactam	10.0 (5)	90.0 (45)
Cefepime	14.0 (7)	86.0 (43)
Ceftazidime	10.0 (5)	90.0 (45)
Amikacin	52.0 (26)	48.0 (24)
Ciprofloxacin	22.0 (11)	78.0 (39)
**Ceftazidime resistant, n = 149**		
Piperacillin–tazobactam	36.2 (54)	63.8 (95)
Cefepime	17.4 (26)	82.6 (123)
Meropenem	69.8 (104)	30.2 (45)
Amikacin	78.5 (117)	21.5 (32)
Ciprofloxacin	36.2 (54)	63.8 (95)
**Amikacin resistant, n = 38**		
Piperacillin–tazobactam	21.1 (8)	78.9 (30)
Cefepime	13.2 (5)	86.8 (33)
Meropenem	36.8 (14)	63.2 (24)
Ceftazidime	15.8 (6)	84.2 (32)
Ciprofloxacin	18.4 (7)	81.6 (31)
**Ciprofloxacin resistant, n = 119**		
Piperacillin–tazobactam	38.7 (46)	61.3 (73)
Cefepime	21.8 (26)	78.2 (93)
Meropenem	67.2 (80)	32.8 (39)
Ceftazidime	20.2 (24)	79.8 (95)
Amikacin	73.9 (88)	26.1 (31)

Data are reported as percentages (absolute numbers).

## Data Availability

Data are contained within the article and Supplementary Materials.

## Supplementary Materials

The following supporting information can be downloaded at https://www.mdpi.com/article/10.3390/antibiotics13121160/s1, Table S1: Distribution of 831 Gram-negatives in 797 BSI included in the study on monotherapy vs. combination. Table S2: Antibiotic susceptibility of 797 Gram-negative strains tested for the different combinations. Table S3: Distribution of 394 isolated pathogens in 382 BSI included in the study for escalation antibiogram.
