# Supplementary material for: Assessing the Theoretical Efficacy of Combination Therapy Against Gram-Negative Infections in Neutropenic Pediatric Cancer Patients: Insights from the Statistical Analysis of Survey Data
Source: Antibiotics (Basel). 2024 Dec 2;13(12):1160. doi: 10.3390/antibiotics13121160 (PMC11672620; doi:10.3390/antibiotics13121160)
Supplement: Supplementary file 1 [file antibiotics-13-01160-s001.zip › antibiotics-3107134-supplementary.pdf]

Supplementary materials

<u>Supplementary table S1</u>: Distribution of 831 Gram-negatives in 797 BSI included in the study on monotherapy vs. combination.

Pathogen	Number of strains
**Enterobacterales**	
Escherichia coli	264
Klebsiella pneumoniae	165
Enterobacter spp	100
Klebsiella oxytoca	16
Serratia marcescens	3
Pantoea agglomerans	3
Pantoea spp	2
Citrobacter koseri	2
Salmonella enteritidis	2
Citrobacter brakii	1
Escherichia fergusonii	1
Leclercia adenocarboxylata	1
Morganella morganii	1
Proteus mirabilis	1
Proteus vulgaris group	1
Raoultella ornithinolytica	1
Salmonella spp	1
Klebsiella ozenae	1
**Glucose non-fermenting Gram-negatives**	
Pseudomonas aeruginosa	132
Stenotrophomonas maltophilia	32
Acinetobacter baumannii complex	28
Acinetobacter spp	15
Burkholderia cepacia	7
Pseudomonas putida	3
Pseudomonas stutzeri	3
Moraxella catarrhalis	3
Pseudomonas fluorescens	2
Pseudomonas luleola	2
Moraxella osloensis	2
Achromobacter xylosoxidans	2
Pseudomonas luteola	1
Burkholderia phenazinium	1

Moraxella non-*liquefaciens*	1
Moraxella spp	1
Ralstonia insidiosa	1
Ralstonia picketii	1
Sphingobacterium spp	1
Sphingomonas paucimobilis	1
other	
Capnocytophaga spp	3
Ochrobactrum anthropi	3
Capnocytophaga sputigena	1
Not further speciated	19

<u>Supplementary table S2</u>: Antibiotic susceptibility of 797 Gram-negative strains tested for the different combinations

	Resistant	Susceptible	Resistant to 1 antibiotic of the combination	Not tested
Piperacillin-tazobactam	21.8 (174)	50.9 (406)	-	27.3 (217)
Cefepime	25.8 (206)	41.9 (334)	-	32.3 (257)
Meropenem	9.0 (72)	74.0 (590)	-	17.0 (135)
Ceftazidime	29.5 (235)	46.0 (367)	-	24.5 (195)
Amikacin	7.5 (60)	61.9 (493)	-	30.6 (244)
Piperacillin-tazobactam + amikacin	4.5 (36)*	35.6 (284) *	20.3 (162)	39.6 (315)^
Cefepime + amikacin	6.0 (48)*	38.4 (306) *	21.3 (170)	34.3 (273)^
Meropenem + amikacin	4.5 (36)*	57.0 (454) *	7.5 (60)	31.0 (247)^
Ceftazidime + amikacin	6.0 (48)*	37.3 (297) *	25.0 (199)	31.7 (253)^

Data are reported as percentages (absolute numbers)

*Resistant or susceptible to both antibiotics of the combination

^ Not tested or susceptible to 1 antibiotic of the combination

<u>Supplementary table S3</u>: Distribution of 394 isolated pathogens in 382 BSI included in the study for escalation antibiogram

**Pathogen**	**Number of strains**
Enterobacterales	
Escherichia coli	133
Klebsiella pneumoniae	89
Enterobacter spp	45
Klebsiella oxytoca	9
Serratia marcescens	1
Pantoea agglomerans	1
Citrobacter koseri	2
Citrobacter brakii	1
Leclercia adenocarboxylata	1
Other glucose non-fermenting Gram-negatives	
Pseudomonas aeruginosa	82
Stenotrophomonas maltophilia	1
Acinetobacter baumannii complex	13
Acinetobacter spp	5
Pseudomonas putida	1
Pseudomonas stutzeri	1
Pseudomonas fluorescens	1
Pseudomonas luleola	1
Achromobacter xylosoxidans	1
Ralstonia insidiosa	1
Ralstonia picketii	1
Sphingobacterium spp	1
Other	
Ochrobactrum anthropi	1
Not further speciated	2
